# Editorial Comment: Factors associated with utilization of neoadjuvant chemotherapy in charlson comorbidity zero non-metastatic muscle-invasive bladder cancer patients

**DOI:** 10.1590/S1677-5538.IBJU.2020.0594.1

**Published:** 2021-07-20

**Authors:** Fernando Korkes

**Affiliations:** 1 Faculdade de Medicina do ABC Disciplina de Urologia Santo AndréSP Brasil Disciplina de Urologia, Faculdade de Medicina do ABC, Santo André, SP, Brasil

## COMMENT

The authors have demonstrated an exciting evolution of neoadjuvant chemotherapy (NAC) trends after radical cystectomy (RC) in the USA in the present study (1). During the last two decades, NAC has proved to bring benefits to these patients. It becomes clear when we follow the National Cancer Database during this period that implementing NAC is not easy, and this practice is growing slowly during the last fifteen years. Including a medical oncologist in the multidisciplinary care of these patients is important and to perform an adequate treatment. Current guidelines recommend NAC with cisplatin-based regimens (2). However, it is not uncommon to encounter patients receiving NAC based on carboplatin, which is not recommended by these guidelines (2). It is important to state that for patients not suitable for cisplatin NAC, RC is still mainstream (3). In the present study, the authors evaluated patients with CCI of zero. Even though we should expect healthy patients, there always could be some contra-indications of cisplatin-based regimens in this CCI zero population. For instance, patients with hearing loss, high ECOG performance score, neuropathy, or slightly reduced renal function can still be CCI of zero but cisplatin-ineligible. For this reason, it is not expected that all CCI zero patients undergo NAC. However, for every patient with muscle-invasive bladder cancer, NAC has to be considered during treatment decisions.
